# Gender Differences in Individuals at High-Risk of Psychosis: A Comprehensive Literature Review

**DOI:** 10.1155/2015/430735

**Published:** 2015-01-01

**Authors:** Ana Barajas, Susana Ochoa, Jordi E. Obiols, Lluís Lalucat-Jo

**Affiliations:** ^1^Department of Research, Centre d'Higiene Mental Les Corts, Network Group for Research in Women's Mental Health (NGRWMH), 08029 Barcelona, Spain; ^2^Departament de Psicologia Clínica i de la Salut, Facultat de Psicologia, Universitat Autònoma de Barcelona, Bellaterra, 08193 Cerdanyola del Vallès, Spain; ^3^Parc Sanitari Sant Joan de Déu, Centro de Investigación Biomédica en Red de Salud Mental (CIBERSAM), Network Group for Research in Women's Mental Health (NGRWMH), Sant Boi de Llobregat, 08830 Barcelona, Spain

## Abstract

*Introduction*. To date, few studies have focused on the characterization of clinical phenomenology regarding gender in population at high-risk of psychosis. This paper is an attempt to summarize the findings found in the scientific literature regarding gender differences in high-risk populations, taking into account parameters studied in populations with schizophrenia and other psychotic disorders, such as incidence, clinical expression, duration of untreated illness (DUI), social functioning, and cognitive impairment prior to full-blown psychosis development. *Method*. Studies were systematically searched in PubMed. Studies using gender variable as a control variable were excluded. 12 studies met inclusion criteria. *Results*. Most of the studies found a differential pattern between women and men as regards clinical, social, and cognitive variables in the prodromal phase, with worse performance in men except in cognitive functioning (more severe negative symptoms, worse social functioning, and longer DUI in men). Similar conversion rates over time were found between men and women. *Conclusions*. Many of the studies analyzed suggest that differences between men and women in the expression of psychosis extend across a continuum, from the subclinical forms of illness to the debut of psychosis. However, the small number of studies and their significant methodological and clinical limitations do not allow for firm conclusions.

## 1. Introduction

Gender differences in the clinical expression and outcome of schizophrenia and first-episode psychosis have long been recognized in the literature on schizophrenia and other psychoses [1–5]. Thereby, men and women experience psychosis differently and often require different intervention methods regarding doses and/or types of medications, staging of interventions, and array of treatments offered [6–8]. These studies have meant an advance in the adaptation and improvement of therapeutic strategies targeting this population. Taking into account gender differences found from the dimensional perspective of psychosis, it would be reasonable to consider different expression of the illness from early phases of psychosis, even before the illness appears. Then, learning whether gender differences in clinical presentation and general functioning are present prior to the development of psychosis is an issue of high priority because it could have implications in intervention strategies, making it possible to maximize the impact of these treatments taking gender into account.

In the last two decades, scientific and clinical interest has been focused on early identification of psychosis and intervening as soon as possible to improve the illness prognosis. In this sense, new intervention strategies have focused on people with signs of incipient psychosis, presenting with potentially prodromal symptoms, who have principally been categorized into three clusters of subjects: young people with attenuated positive symptoms, as revealed by dedicated interviews [9]; people with diagnosable transient psychotic symptoms, not stabilized in a syndrome yet [10, 11]; and a third category of people with genetic risk (first degree relatives of subjects with psychosis), or meeting the criteria for schizotypal personality disorder, who are showing symptoms of deterioration [12]. This clinical syndrome has been termed an at-risk mental state [13], and operationalized criteria—the ultra-high risk (UHR) [14] or clinical high-risk (HR) criteria [15]—have been developed to identify the syndrome. These criteria have been adopted and adapted in a number of other settings around the world [9]. However, this new approach has not taken into account the gender variable, due in part to the lack of research on this topic. To date, only a few studies have focused on the characterization of clinical phenomenology regarding gender in population at high-risk of psychosis.

This paper attempts to summarize the findings in the scientific literature regarding gender differences in high-risk populations, taking into account parameters studied in populations with schizophrenia and other psychotic disorders, such as incidence, clinical expression, duration of untreated illness (DUI), social functioning, and cognitive impairment prior to full-blown psychosis development.

In this review we will try to discuss the following hypothesis: if gender-related factors are equally meaningful over the entire psychosis continuum, it is reasonable to expect that gender differences could also be already identified in subclinical psychosis. It would then be presumed that the same (continuous) development pattern by gender exists from gestation of psychosis until the psychosis threshold. Alternatively, if the hypothesized gender differences are not found, we would have to assume that there are different patterns in how symptoms develop in men and women, with exponential and differential effects starting only when the psychosis threshold is reached.

## 2. Method

### 2.1. Inclusion and Exclusion Criteria

Studies analyzing gender differences in high-risk populations for psychosis were considered. Those examining determined outcome measures, such as incidence, clinical expression, duration of untreated illness (DUI), social functioning, and cognitive impairment, were selected. Studies using gender variable as a control variable, in which the main aim was not to examine gender differences in the parameters above indicated, were excluded.

### 2.2. Search Strategy and Study Selection

PubMed was consulted twice using the following search terms and Boolean operators up to May 31, 2014: (1) a specific search using* high-risk *AND* psychosis *AND (*gender* OR* sex*) AND (*epidemiology* OR* incidence* OR* transition* OR* symptoms* OR* clinical* OR* duration of untreated illness* OR* DUI* OR* social functioning* OR* disability* OR* cognitive functioning* OR* cognitive impairment* OR* cognition* OR* neurocognition*), and (2) a general search using* high risk *AND* psychosis *AND (*gender* OR* sex*). No additional filters regarding the publication date of the articles were used.

The general and specific PubMed searches together generated 258 hits. After removing double hits and screening title and abstract according to the inclusion and exclusion criteria mentioned above, 20 potentially relevant papers were retrieved for more detailed evaluation. These studies were screened on meeting the inclusion criteria and subsequently 12 studies were excluded after reading the full texts, due to irrelevant subject, qualitative methodology, or descriptive nature. Four studies cited in articles selected across PubMed, fulfilling inclusion criteria, were also included. Finally, 12 studies met the inclusion criteria, which are included in Table 1 and in the references list of this paper [16–27].

### 2.3. Data Extraction

Eligible studies were independently screened by two researchers (S. Ochoa and A. Barajas) to verify the fulfillment of the criteria. The following variables were extracted to generate Table 1: (1) author and publication data, (2) participant characteristics, (3) outcome variables, and (4) main findings. Studies were grouped according to whether outcome measures were related to the following topics: epidemiology, clinical expression, social functioning, and cognitive functioning. For each topic, a general conclusion was extracted in order to summarize the main findings about gender differences in high-risk populations for psychosis.

## 3. Results

### 3.1. Epidemiology: Incidence of Transition to Psychosis

Historically, gender differences in schizophrenia cases have been associated with almost all aspects of the disease, including incidence and prevalence ([28–32], see Table 2). The epidemiological characteristics of a disorder can provide important clues in the search for etiology and are essential in the development of evidence-based treatment models. No gender differences in prevalence of schizophrenia have been found in recent studies. Nevertheless, the most replicated result of incidence studies is a higher rate in males versus females in patients with schizophrenia or/and schizophrenia-like psychosis. It is possible that the stricter the diagnostic criteria for schizophrenia, the greater the exclusion for women, resulting in a higher proportion of men diagnosed. However, other authors have attributed this discrepancy in gender differences between incidence and prevalence to clinical variables: a higher suicide rate in men with schizophrenia compared with women [5] or a higher trend to briefer episodes of psychosis, with more complete resolution, in women [33].

According to the continuum hypothesis [34], gender-related factors as identified in full-blown psychosis would be equally meaningful over the entire psychosis continuum and we should expect that “true” gender differences could also be identified in subclinical psychosis. However, to date it is unknown whether gender differences in the epidemiology of schizophrenia extend to those subjects who are at high-risk of developing psychosis. Only a few studies have addressed this question, and they have presented inconsistent results. In a recent study, as part of the North American Prodrome Longitudinal Study (NAPLS) [21], gender differences in the antecedents and course of the prodrome to psychosis in clinical high-risk adolescents and young adults were analyzed. No gender differences were found in conversion rates at 2.5 years follow-up (26.5% women; 24.5% men). In the same sense, Lemos-Giráldez et al. [18]. found that the conversion rate to psychosis was 22.95% in the three-year follow-up period without statistical gender differences (22.5% men versus 23.8% women). In addition, in a study with help-seeking adolescents at ultrahigh risk (UHR) for psychosis, Ziermans et al. [20] showed that at the end of the follow-up period (2 years) 15.6% of UHR adolescents had experienced a psychotic transition, with a higher proportion of men. Furthermore, Nordentoft et al. [17] found that, among young adults with a diagnosis of schizotypal disorder, men had a fourfold greater risk for conversion to schizophrenia one-year after enrollment when compared to women. However, the findings of this study may not be directly comparable to the entire UHR population, which includes a wider definition of psychosis risk. To date, it is unclear if at-risk-mental-state in men is associated with a higher risk of progression to schizophrenia than in women. Amminger et al. [16] found that the rate of conversion to nonaffective psychosis was not higher among men than women. They explained this result in relation to the greater weight of positive psychotic symptoms in the UHR criteria definition, which might be more effective in detecting “true” prodromal cases among women than men because they focus on positive attenuated symptoms, with minimal attention to negative symptoms. This interpretation is supported by Maric et al. [35], who reported that, in a general population sample, subclinical positive psychotic symptoms were more prevalent in women, whilst subclinical negative psychotic symptoms were more prevalent in men. Some epidemiological investigations that took a more general approach concerning subclinical psychosis have detected no gender differences [36, 37]. Van Os et al. [34] found slightly increased odds ratios for men in a meta-analysis of epidemiological studies focusing on subclinical psychosis. These results referred only to prevalence rates whereas the incidence rate in that meta-analysis was minimally higher for women. Spauwen et al. [38] analyzed a representative Dutch population sample (aged 17 to 28), with their main focus being on possible gender differences before and after the age of 21. They found that the incidence of subclinical psychotic experiences was higher in men aged 17 to 21 but then became similar to that of women when those men reached 22 to 28 years of age.

On the other hand, Goldstein et al. [19] demonstrated that there are sex-specific patterns of transmission of psychosis. Among fathers with psychoses most offspring who developed psychosis were female (15.2% females versus 3.1% males); in contrast, among mothers with psychosis 18.8% of their male offspring developed psychosis compared with 9.5% of their daughters. So that risk of psychosis among the offspring of parents with psychosis was dependent on the gender variable. These results show the importance of considering sexual differentiation in the concept of psychosis risk.

Finally, it is important to note that more women than men seek help for psychological or medical problems [39]. Taking this into account, additional strategies of detection and different criteria are needed to determine real incidence rates in risk mental states of psychosis.

The disparity found between these findings, as in the schizophrenia studies, could be due to clinical and/or methodological factors: the lack of consensus among the studies in defining a patient at high-risk for psychosis, as well as the lack of differential detection strategies according to gender, could be some possible explanations. In this sense, in epidemiological terms, it is not possible to confirm continuity in gender differences between subthreshold psychosis and frank psychosis.

In relation to the lack of consensus on the definition of risk criteria for psychosis, they could be based on expression differential between women and men as regards clinical, social, and cognitive variables in the prodromal phase. We will now turn to a summary of studies that have analyzed the differences in clinical, functional, and cognitive expression by gender in individuals at risk of psychosis.

### 3.2. Clinical Expression and DUI

Gender differences in symptom expression have important implications for several reasons. For example, symptom presentation likely plays an important role in determining treatment regimens and understanding gender differences in treatment response. To date, the results in psychosis spectrum disorder studies regarding this area are inconclusive ([40–47], see Table 2). A number of factors may account for this, including medication status (higher doses of typical antipsychotics contributing to negative symptomatology), diagnostic stringency (use of stricter criteria excluding women with affective symptoms), age at onset (negative symptoms are more prominent in younger men than in younger women), and sampling bias (inadequate sample size or overrepresentation of men). Nevertheless, the most replicated findings, in samples of patients with psychotic spectrum disorders, suggest that affective symptoms are more common in women while negative symptoms tend to be more predominant in men. These findings have also been found in some studies analyzing gender differences in UHR samples. Willhite et al. [22] found that, prior to the expression of full-blown psychosis, young men were rated as having more severe negative symptoms than women when baseline and follow-up time points (6- and 12-month) were jointly considered. This result is consistent with the large literature in the field, in both studies of individuals at-risk for psychosis [23] and studies about psychotic disorders spectrum [1, 48, 49], indicating that the differences between men and women in clinical presentation extend across the continuum of psychosis. Men with UHR for psychosis had more “typical” symptoms of schizophrenia than women. However, there was no effect of gender for ratings on the other symptom dimensions using the Structured Interview for Prodromal Syndromes (SIPS). In another study analyzing individuals with UHR for psychosis by gender [16] it was found that female gender was one of the independent significant predictors of affective psychosis, which is in accordance with the higher prevalence of affective disorders in women [50]. These findings suggest that underlying gender differences may predate the onset of psychosis. This could reflect the fact that men and women are vulnerable to different “types” of psychotic disorders or that psychosis develops differently in men and women. In contrast, other studies of individuals with UHR for psychosis did not find gender differences in the expression of symptoms [18, 24]. As already mentioned in the previous section, methodological and clinical variables could influence the discrepancy of the results found, similarly to what occurs in schizophrenia and first-episode psychosis studies.

In relation to onset of illness, in the ABC first-episode sample with a broad definition of schizophrenia, Häfner [51], showed that the disorder manifests itself clearly later in women than men, from the first sign of the illness. Women's mean age at first symptom was 25.4 years, 2.9 years higher than men's age. On the other hand, women in general are more help seeking and have a more positive attitude towards taking medication than men [52]. Further, women tend to express their emotions more readily and behave in a relatively unobtrusive way. Thus, women patients may be more likely to be misdiagnosed, for example, with a mood disorder [53]. Also, it is widely accepted that women have better access to social networks and social support [54]. This behaviour pattern together with a later onset of first symptoms of illness could indicate a shorter duration of untreated illness in women. However, Cocchi et al. [24] and previous studies [16, 18] with UHR samples did not find significant differences by gender in DUI, which was shorter in women than in men in most samples. This discrepancy may be a consequence of faster and longer deterioration in men when symptoms arise [18]. Additionally, in the general population age-related gender differences in maturational processes have been observed, in particular, a significantly greater loss of cerebral grey matter in boys compared to girls [55], which may represent the underlying mechanism of men showing an earlier age of onset of psychosis than women in subclinical samples. An alternative explanation may be found in the differential exposure to estrogens which may play a protective role by decreasing the risk for and the severity of psychotic disorders in women [56]. Despite the results about DUI being replicated in UHR samples, we must take into account that a high percentage of the sample will not transition to psychosis. So the implications of these findings in the continuum of psychosis should be treated with caution. These findings should be compared with studies about UHR individuals who convert to psychosis.

Regarding the association between clinical symptoms and social functioning found in psychotic disorders [57, 58], there are studies with UHR samples which confirm that this association is already present before the onset of psychosis [22, 23]. Because UHR patients may experience different combinations of symptoms according to gender these differences may contribute to different functional outcomes. Based on these results, developing targeted intervention to decrease the severity of symptoms would improve the general functioning and quality of life for patients.

### 3.3. Social Functioning

In general, psychosis spectrum disorder studies analyzing premorbid and social functioning show better performance in women ([45, 59–63], see Table 2). We have not found studies reporting worse social functioning in women. However, there are studies reflecting the absence of gender differences in this area ([47, 64], see Table 2). Methodological issues might explain these discrepancies (i.e., small sample size, lack of sample-gender representativeness, or measures used to assess social functioning). Similar conclusions can be drawn from studies with patients at high-risk for psychosis. Willhite et al. [22] investigated gender differences in functioning and social support in individuals at UHR for developing a psychotic disorder, showing that men had marginally lower functioning than women over the three time points (at baseline, 6- and 12-month follow-up). Differences in other psychosocial factors may also contribute to better functioning in women UHR patients. Women reported higher levels of social support at baseline: women were more likely to say that their friends and family members “appreciate them” and that they feel they can “open up” to their friends and family members, while men report marginally higher levels of criticism than women. These results would support the importance of psychosocial interventions for this population. However, subsequently, mixed results have been obtained, from studies that contradict these results [18] as well as those that confirm them [25].

In relation to premorbid functioning, most studies in psychotic disorder samples have found gender differences, this being worse in men than in women [44, 46, 65]. Contrary to expectation, the NAPLS study [21] found that early childhood academic, social, and total adjustments were comparable by gender. It is possible that impairment at this age may be too subtle to be detected, being more evident from adolescence.

On the other hand, there are suggestions that deficits in social functioning are often predictors of later social functioning [66]. Besides, prior findings have indicated that social and role (school/work) functioning are key predictors of conversion among UHR youth [67, 68]. Therefore, prevention strategies could be improved with a more comprehensive approach that involves developmentally earlier functional deficits. However, it has been suggested that social functioning as a predictor of psychosis onset may be stronger for men than women [22, 26]. These findings are corroborated in Walder et al. [21]; specifically, poorer baseline social functioning and positive prodromal symptoms predict greater conversion risk among men. So, to understand the early development of psychosis it is essential to consider sexually differentiated predictors. It may be helpful to improve risk identification using algorithms that take into account the gender variable. To date a global assessment of functioning (GAF) score of 50 or below has been used in the risk criteria, but gender variable was not considered.

There is some agreement among the results found in the scientific literature about full-blown psychosis and the UHR studies regarding social and role functioning. These results support the continuum hypothesis in the development of psychosis, taking into account a differential expression of social functioning according to gender from premorbid phases, which is worse in men. Nevertheless, broader samples with more equal distribution by gender are essential for more rigorous investigation.

### 3.4. Cognitive Functioning

Although studies analyzing cognitive function in patients with psychosis spectrum disorders have reported gender differences on neuropsychological testing ([69–72], see Table 2), these differences have not been tested in all studies ([73, 74], see Table 2) and their nature is controversial. As indicated in previous sections, these discrepancies could be partly related to methodological issues. Among studies showing gender differences, the finding most often replicated indicates higher levels of cognitive functioning in women.

Over the past several years, under the assumption that the prodromal period is marked by disruptions in the normal brain maturation processes having an impact on neurocognition, there has been an accumulation of data examining cognition in those at elevated risk for psychosis [75–77], which have confirmed that cognitive deficits are already present before the first episode. These studies report widespread cognitive deficits intermediate to healthy control and first-episode psychosis samples. However, these studies have several methodological limitations (i.e., small samples, lack of power to detect differences, and a limited longitudinal framework, among others) that make it difficult to generalize the results. In this sense more research about cognition functioning is required, also taking into account gender factor from the incipient phases of psychosis. To date, scarce research has been performed in this direction. To our knowledge, the study of Walder et al. [27] is the only one to consider this issue as a main objective. It is a longitudinal study of 37 adolescents at high-risk for psychosis, which showed that women who convert performed worse on several neurocognitive measures than same-gender subjects who do not convert. There were no significant differences between men converters and nonconverters. In the group who converts, women showed worse performance than their high-risk male counterparts on a measure of verbal memory, unlike the results found with schizophrenia patients [71]. The low number of women converters did not allow for valid conclusions on gender differences. Nevertheless, these results suggest the importance of considering sexually differentiated patterns of cognitive decline in prodromal individuals. Furthermore, sexually differentiated neurohormonal fluctuations are present during adolescence and early adulthood and may play an integral role in the transition to psychosis [78].

More studies are needed to explore a possible continuity of cognitive decline according to gender from subthreshold phases of psychosis up to its onset. Future longitudinal research, overcoming the previous methodological limitations and aimed at tracking at-risk cohorts from premorbid periods until clinical high-risk to psychosis onset, will help clarify the existence of neurocognitive profiles in predicting conversion risk based on gender differences.

## 4. Conclusions

In conclusion, although the extent of gender differences in individuals with UHR for psychosis is reduced mainly due to the small number of studies published to date and their limitations (see Section 5), in this section we will try to summarize the most relevant findings and their clinical and research implications.

- Inconsistent results were found in relation to transition to psychosis: some studies did not show gender differences and others indicated a greater risk for conversion to psychosis in men. It might be suggested that differential precipitating factors exist according to gender which are involved in conversion to psychosis and their identification should be useful in clinical practice.
- Men at-risk for psychosis have more severe negative symptoms than women before full-blown psychosis, being more difficult to detect them across current risk criteria for psychosis focused on positive attenuated symptoms. In addition, developing targeted intervention to decrease the severity of negative symptoms during prodromal phase, more severe in men, would improve the general functioning and quality of life for patients since earlier phases of psychosis.
- Female gender is one of the independent significant predictors of affective psychosis. It highlights the importance of considering sexually differentiated high-risk criteria to improve the identification of possible risk cases with different diagnoses in the continuum of psychosis.
- Significant gender differences have not been found in DUI, although it is shorter in women than in men in most of the studies. It is likely that more women seek help for psychological or medical problems than men. Additional detection strategies, especially targeted at males, should be developed, not only to improve the quality of research but also above all to prevent the development of more severe forms of the disease.
- Men show lower functioning and social support than women before full-blown psychosis. Also, social functioning is a stronger predictor of psychosis onset in men than women. Psychosocial interventions targeted at this population would be helpful to improve the prognosis of the illness.
- The limited scientific evidence about cognitive impairment in prodromal phase according to gender has indicated a differential sex effect that varies by risk status. However, the lack of studies does not allow us to generalize from the results. It is necessary to broaden our knowledge in this area and so be able to implement the findings in clinical practice.

In summary, many of the studies analyzed suggest that differences between men and women in the expression of psychosis extend across a continuum, from the subclinical forms of illness to the debut of psychosis, mainly in aspects of clinical expression (such as more negative symptoms in men) and social functioning (such as premorbid and psychosocial functioning, worse in men). However, the small number of studies and their significant methodological and clinical limitations do not allow for firm conclusions.

## 5. Limitations and Future Directions

We must be cautious when considering the extracted conclusions of this comprehensive review about gender differences in individuals at high-risk for psychosis due to the limitations of the studies analyzed. The main clinical and methodological limitations of the studies included in this review are the following: (a) the limited sample size with unequal distribution of participants at baseline by gender, (b) a strict age range, (c) the exclusion of people with substance dependence, (d) the lack of standardized instruments to measure some variables (e.g., DUI), (e) the use of diagnostic criteria for the UHR which require the presence of symptoms in excess, and (f) the large attrition rates. Taking into account these limitations, it is possible that the samples of these studies are not representative of the population of all UHR cases and may obscure or reduce the extent of gender-related differences found. Also, the methodological variability of these studies limits the generalizability of their findings. Nevertheless, something to keep in mind is that gender differences might be less evident in UHR samples than in samples of people diagnosed with psychotic disorders because only a minority of UHR people develops full-blown psychosis [79].

More research is needed to overcome the limitations mentioned in order to deepen our knowledge about this topic. Future researchers should be focused on (a) improving detection algorithms for risk of psychosis, due to differential expression of subclinical forms of psychosis according to gender; according to the results found, there is a need to include negative symptoms in the risk criteria, which may help to identify more young men at risk for psychosis; (b) analyzing other variables or risk factors by gender such as substance use, family history, stress, or obstetric complications; (c) using gender as a covariable in future research about effectiveness of interventions in prodromal phase; (d) studying gender differences in the precipitating factor for psychosis; (e) analyzing different pattern of cognitive impairment by gender; and (f) identifying protector factors delaying conversion by gender. Further elucidation of differential pattern by gender during the prodrome to psychosis is critical to understanding illness etiology and generating more powerful predictive models that would be maximally sensitive and specific. This may aid in development of individually tailored treatments, with consideration of the effects of gender, which may target different neurobiological systems and/or use alternative cognitive/behavioral approaches with optimal effect. Gender-sensitive services are a first step toward individual-specific personalized care, as men and women may differentially benefit from certain approaches to intervention.

## Figures and Tables

**Table 1:** Overview of gender differences in high-risk population for psychosis, according to outcome measures: results and main conclusions.

	Study	Sample characteristics	Outcome measures	Key finding	Main conclusion
Epidemiology	Amminger et al., 2006 [16]	86 individuals considered to be at UHR for schizophrenia	Transition to psychosis	The rate of conversion to nonaffective psychosis was not higher among males than females. Female gender was a significant predictor of conversion to affective psychosis at 2-year follow-up.	Inconsistent results were found: some studies did not show gender differences and others indicated a greater risk for conversion to psychosis in men. More studies analyzing gender differences regarding transition to psychosis are needed.
	Nordentoft et al., 2006 [17]	79 patients diagnosed with schizotypal disorder	Transition to psychosis	Males versus females had a fourfold risk [RR: 4.47, CI: 1.30–15.33] for conversion to schizophrenia at 1-year follow-up.	
	Lemos-Giráldez et al., 2009 [18]	61 participants meeting criteria for UHR of psychosis	Transition to psychosis	The conversion rate to psychosis was 22.95% in the three-year follow-up period without gender statistical gender differences (22.5% men versus 23.8% women).	
	Goldstein et al., 2011 [19]	159 parents with psychoses and 114 comparable, healthy control parents. 203 HR and 147 control offspring.	Transmission to psychosis	Risk of psychosis in offspring was a function of the sex of parent and offspring. The male : female ratio for affected offspring differed significantly between affected mothers and fathers.	
	Ziermans et al., 2011 [20]	72 adolescents putatively at UHR for psychosis	Transition to psychosis	15.6% of UHR adolescents converted to psychosis at 2-year follow-up, with a higher proportion of men (13.8%).	
	Walder et al., 2013 [21]	276 CHR NAPLS participants	Transition to psychosis	CHR participants who converted to psychosis (70, 34%) at some point over a 2.5-year follow-up period did not significantly differ in gender distribution.	

Clinical expression	Willhite et al., 2008 [22]	68 UHR patients assessed at baseline, six and twelve months later	Symptoms	Males appeared to have more severe negative symptoms when data from all three time points were examined.	Differences by gender were modest or absent under many variables that were classified as typical of schizophrenia in the past. More negative symptoms in men versus females are the most replicated result in some of the CHR population studies, as in studies of patients with a psychotic spectrum disorder.
	Lemos-Giráldez et al., 2009 [18]	61 participants meeting criteria for UHR of psychosis	Symptoms and recovery pattern	No gender differences in symptom or functioning levels at the three follow-up time points (at baseline, at 1-year and at 3-year follow-up) were found. Males experienced faster and longer deterioration when psychotic symptoms arise.	
	Corcoran et al., 2011 [23]	56 young people at CHR for psychosis	Negative symptoms	As for gender, male patients only had significantly more negative symptoms than females.	
	Cocchi et al., 2014 [24]	106 patients at UHR of psychosis	Age at onset and DUI	No gender differences were found in age of onset, DUI, symptom severity, or level of functioning.	

Social functioning	Willhite et al., 2008 [22]	68 UHR patients assessed at baseline, six and twelve months later.	Functioning and social support	Males were rated as having marginally lower functioning than females over the three follow-up time points. Males reported less positive social support than their female counterparts and felt they received marginally more criticism than females.	Most studies indicated gender differences in premorbid and psychosocial functioning, being worse in men. Social dysfunction in adolescence might be a good predictor of transition to psychosis.
	Lemos-Giráldez et al., 2009 [18]	61 participants meeting criteria for UHR of psychosis	Social functioning	No gender differences in functioning levels at the three follow-up time points (at baseline, at 1-year and at 3-year follow-up) were found.	
	Salokangas et al., 2013 [25]	244 young help-seeking CHR patients	Psychosocial state	At baseline, males' psychosocial situation was poorer than that of females, but at follow-up there was no longer any gender difference in psychosocial outcome. There was also no gender difference in global outcome.	
	Tarbox et al., 2013 [26]	270 CHR individuals in the NAPLS	Premorbid functioning	In the full CHR sample, male participants had worse social functioning in early and late adolescence. Early adolescent social dysfunction significantly predicted conversion to psychosis.	
	Walder et al., 2013 [21]	276 CHR NAPLS participants	Premorbid adjustment and social and role functioning	Baseline social and role functioning are more impaired in CHR males than females (though not in early childhood adjustment).	

Cognitive functioning	Walder et al., 2008 [27]	37 youth at HR for psychosis	Neurocognitive performance	AHRP+ females performed worse than same-gender AHRP− on several neurocognitive measures. There were no significant differences between male converters and nonconverters. AHRP− males performed worse than their HR female counterparts on a variety of neurocognitive measures. AHRP+ females performed worse than their HR male counterparts on a measure of verbal memory.	Neurocognitive performance in CHR shows a differential gender effect that varies by risk status. It suggests the importance of considering sexually differentiated patterns of cognitive decline in prodromal subjects.

CHR: clinical high-risk; NAPLS: North American Prodrome Longitudinal Study; HR: high-risk; UHR: ultrahigh risk; RR: relative risk; CI: confidence interval; AHRP+: at high-risk for psychosis who converted; AHRP−: at high-risk for psychosis who did not convert.

**Table 2:** Overview of gender differences in psychosis spectrum disorder studies: controversial results and main conclusions.

	Study	Sample characteristics^*^	Outcome measures	Key finding^**^	Main conclusion
Epidemiology	Castle et al., 1993 [28]	470 first-contact patients with nonaffective functional psychosis	Incidence	The ratio of male to female incidence rates rose progressively when RDC (1.2), DSM-III-R (1.3), DSM-III (2.2), and Feighner (2.5) criteria for schizophrenia were applied. Schizophrenia was most common in young males and least common in older males, with females occupying an intermediate position.	Gender differences in incidence rates were found in patients with schizophrenia and schizophrenia-like psychosis, being higher in males. However, no gender differences were found in terms of prevalence. These results may depend on the stringency of the diagnostic criteria applied (i.e., age cut-off or a narrower definition of schizophrenia in terms of symptomatology and duration of symptoms): the broader the criteria the less significant the gender differences in incidence or prevalence.
	Aleman et al., 2003 [29]	38 publications on “incidence of schizophrenia” published during the period between January 1980 and September 2001	Incidence	The incidence risk ratios for men developing schizophrenia relative to women were 1.42 (95% [CI], 1.30–1.56) when all studies were included in the analysis (49 effect sizes). This risk remained significantly higher in men after controlling for potentially confounding factors (i.e. age bias, criterion bias, and hospital bias).	
	Saha et al., 2005 [30]	A total of 1,721 prevalence estimates from 188 studies	Prevalence	No significant differences in prevalence of schizophrenia were found between males and females. For the male: female estimate ratio (based on 57 ratios), the median value was 1.11, and the 10% and 90% quantiles were 0.50 to 1.70.	
	Perälä et al., 2007 [31]	248 subjects, 30 years or older, with a diagnosis of any psychotic disorder	LTP	The LTP was 3.06% for any psychotic disorder, and regarding gender it was 3.11% for men vs. 3.01% for women. In particular, in relation to schizophrenia diagnosis no gender differences were observed in the prevalence data.	
	McGrath et al., 2008 [32]	For the incidence analysis, 158 studies that generated 1,458 rates For the prevalence analysis, 188 studies that provided 1,721 prevalence estimates	Incidence and prevalence	The distribution of incidence rates differed significantly between males and females, and the median (10, 90 percent quantiles) rate ratio for male : female estimates was 1.4 (0.9, 2.4). The gender difference identified in the incidence rates is not reflected in prevalence estimates (the median lifetime prevalence estimates for males were 3.7 per 1,000 vs. 3.8 per 1,000 for females)	

Clinical expression	Lindström and Von Knorring, 1994 [40]	140 patients with schizophrenic syndromes	Positive, negative, excited, anxious/depressive, and cognitive symptoms	No gender differences were found in the 5-factor model of schizophrenia, including positive, negative, excited, anxious/depressive and cognitive factors.	No consensus exists regarding gender differences in the clinical expression of psychosis. This could be due to clinical and/or methodological factors. Studies showing gender differences obtained discordant results. However, the most replicated findings suggest that mood symptoms are common in women with schizophrenia or FEP, while negative symptoms tend to be more predominant in men.
	Szymanski et al., 1995 [41]	54 patients with first-episode schizophrenia	Affective and positive symptoms	Females displayed significantly less illogical thinking, but more anxiety, inappropriate affect and bizarre behavior than the men.	
	Gur et al., 1996 [42]	272 patients with schizophrenia	Negative and positive symptoms	A greater severity of negative symptoms in men and identical severity of positive symptoms for men and women in all age groups were observed.	
	Hayashi et al., 2002 [43]	308 patients with DSM-IV schizophrenia	Positive, negative, excited, anxious/depressive, and cognitive symptoms	In a five-factorial structure model of PANSS the factors and component symptoms were common across gender.	
	Morgan et al., 2008 [44]	1090 cases of affective and nonaffective psychosis	Positive, affective, and negative symptoms	In schizophrenia specifically, women were significantly less likely than men to report having hallucinations, delusions or poor concentration currently, and more likely to report at least one serious episode of dysphoria over a lifetime. Within all groups, women were less likely than men to experience negative symptoms.	
	Cotton et al., 2009 [45]	661 patients with first episode psychosis	Depressive symptoms	Females were more likely to have depressive symptoms while males experienced more severe psychopathology.	
	Barajas et al., 2010 [46]	53 consecutive cases with a first psychotic episode	Negative symptoms and insight	In the group of younger patients, men showed more negative symptoms and poorer insight than women. No gender differences were observed in the clinical expression of the episode in the older group of patients.	
	Galderisi et al., 2012 [47]	276 patients with spectrum disorders of schizophrenia	Negative symptoms	Female patients, as compared with males, showed fewer negative symptoms.	

Social functioning	Häfner et al., 1993 [59]	267 first-admitted patients with nonaffective functional psychosis	Premorbid and social and occupational functioning	Males had lower social role functioning than females prior to first admission, and of those patients who achieved adequate role functioning, males had greater deterioration in social and occupational functioning after onset.	Better social functioning appears to be a particular strength of women with psychosis spectrum disorders compared to men. However, not all studies confirm this finding. Methodological issues could explain these discrepancies (i.e., sample size, lack of sample representativeness, and measures used to assess social functioning).
	Andia et al., 1995 [60]	85 outpatients with schizophrenia	Psychosocial functioning	Women exhibited better psychosocial functioning (better educated and more often married, living independently, and employed).	
	Usall et al., 2002 [61]	200 outpatients with schizophrenia (DSM-IV criteria)	General functioning and disability	Gender influenced significantly on DAS (occupational and personal care) and GAF, with men showing worse functioning.	
	Grossman et al., 2008 [62]	97 patients with schizophrenia and other psychotic disorders	Global functioning	Women showed significantly better global functioning at 3 of the 6 follow-ups (the 2-, 7.5-, and 10-year follow-ups).	
	Cotton et al., 2009 [45]	661 patients with first episode psychosis	Premorbid and social functioning	There were no gender differences in premorbid functioning (measured by the GAF). Males experienced lower levels of functioning, being less likely to be working/studying, and more likely to be living with their family.	
	Bottlender et al., 2010 [64]	177 patients with life-time diagnoses belonging to the schizophrenic, schizoaffective, or affective spectrum (ICD-10 criteria)	Social disability	No gender differences were found in social disability using DAS scale.	
	Vila- Rodriguez et al., 2011 [63]	231 community-dwelling individuals with schizophrenia	Social functioning	Women scored higher in social functioning (LSP). While men's social functioning is affected by positive symptoms, women's social functioning is impaired by disorganized symptoms.	
	Galderisi et al., 2012 [47]	295 stabilized patients with schizophrenia, schizoaffective, or delusional disorder	Social functioning	No significant effect of gender was observed on any index of social functioning.	

Cognitive functioning	Goldberg et al., 1995 [73]	Four independent schizophrenic cohorts: two groups of inpatients with chronic courses at a research hospital (*N* = 191), one group of consecutive admissions to a private psychiatric hospital (*N* = 57), and one group of schizophrenic twins from discordant monozygotic pairs (*N* = 20)	Neuropsychological performance	Not one comparison significantly favored women, and few were even significantly different by gender.	The inconsistencies in the results may be due mainly to methodological questions (i.e., samples of convenience including not representative male and female patients, analysis not controlling clinical variables, lack of normal comparison group, or lack of consensus in measures used).
	Lewine et al., 1996 [69]	195 patients with schizophrenia or schizoaffective disorder	Neuropsychological functioning	Women performed significantly more poorly than men in verbal memory, spatial memory, and visual processing.	
	Purcell et al., 1998 [70]	159 patients with schizophrenia or schizoaffective disorder	VIQ and PIQ	Significantly more men than women with schizophrenia exhibited a VIQ > PIQ pattern.	
	Moriarty et al., 2001 [74]	205 geriatric patients with lifelong poor-outcome schizophrenia	Cognitive impairment	No gender differences in cognitive functioning were found.	
	Bozikas et al., 2010 [71]	96 schizophrenia patients	Basic cognitive abilities	The effect of gender was significant for verbal learning and memory, wherein women outperformed men.	
	Vaskinn et al., 2011 [72]	154 participants with schizophrenia and 106 participants with bipolar I disorder	Neurocognitive performance	Women performed better than men for all neuropsychological tests (except attention and working memory).	

^*^Sample characteristics of the participants with a psychotic spectrum disorder ^**^key finding regarding gender differences.

RDC: research diagnostic criteria; DSM: diagnostic and statistical manual of mental disorder; CI: confidence interval; LTP: lifetime prevalence; PANSS: positive and negative syndrome scale; DAS: disability assessment scale; GAF: global assessment functioning; ICD: international classification of diseases; VIQ: substantial verbal intelligence quotient; PIQ: performance intelligence quotient.
